# Continuation of chronic antiplatelet therapy is not associated with increased need for transfusions: a cohort study in critically ill septic patients

**DOI:** 10.1186/s12871-024-02516-7

**Published:** 2024-04-17

**Authors:** Christian Fuchs, Christian S. Scheer, Steffi Wauschkuhn, Marcus Vollmer, Konrad Meissner, Klaus Hahnenkamp, Matthias Gründling, Sixten Selleng, Thomas Thiele, Rainer Borgstedt, Sven-Olaf Kuhn, Sebastian Rehberg, Sean Selim Scholz

**Affiliations:** 1 Department of Anaesthesiology, University Medicine Greifswald, Greifswald, Germany; 2 Department of Psychosomatic Medicine and Psychotherapy, Ernst von Bergmann Hospital, Potsdam, Germany; 3 Institute of Bioinformatics, University Medicine Greifswald, Greifswald, Germany; 4 Department of Anaesthesiology, University Medical Center Göttingen, Göttingen, Germany; 5 Institute of Transfusion Medicine, University Medicine Greifswald, Greifswald, Germany; 6 Department of Anaesthesiology, Intensive Care, Emergency Medicine, Transfusion Medicine and Pain Therapy, Medical School, Protestant Hospital of the Bethel Foundation, Bielefeld University, University Medical Center OWL, Burgsteig 13, 33617 Bielefeld, Germany

**Keywords:** Sepsis, Antiplatelet therapy, Transfusion, RBC, Survival

## Abstract

**Background:**

The decision to maintain or halt antiplatelet medication in septic patients admitted to intensive care units presents a clinical dilemma. This is due to the necessity to balance the benefits of preventing thromboembolic incidents and leveraging anti-inflammatory properties against the increased risk of bleeding.

**Methods:**

This study involves a secondary analysis of data from a prospective cohort study focusing on patients diagnosed with severe sepsis or septic shock. We evaluated the outcomes of 203 patients, examining mortality rates and the requirement for transfusion. The cohort was divided into two groups: those whose antiplatelet therapy was sustained (*n* = 114) and those in whom it was discontinued (*n* = 89). To account for potential biases such as indication for antiplatelet therapy, propensity score matching was employed.

**Results:**

Therapy continuation did not significantly alter transfusion requirements (discontinued vs. continued in matched samples: red blood cell concentrates 51.7% vs. 68.3%, *p* = 0.09; platelet concentrates 21.7% vs. 18.3%, *p* = 0.82; fresh frozen plasma concentrates 38.3% vs. 33.3%, *p* = 0.7). 90-day survival was higher within the continued group (30.0% vs. 70.0%; *p* < 0.001) and the Log-rank test (7-day survivors; *p* = 0.001) as well as Cox regression (both matched samples) suggested an association between continuation of antiplatelet therapy < 7 days and survival (HR: 0.24, 95%-CI 0.10 to 0.63, *p* = 0.004). Sepsis severity expressed by the SOFA score did not differ significantly in matched and unmatched patients (both *p* > 0.05).

**Conclusions:**

The findings suggest that continuing antiplatelet therapy in septic patients admitted to intensive care units could be associated with a significant survival benefit without substantially increasing the need for transfusion. These results highlight the importance of a nuanced approach to managing antiplatelet medication in the context of severe sepsis and septic shock.

**Supplementary Information:**

The online version contains supplementary material available at 10.1186/s12871-024-02516-7.

## Introduction

Antiplatelet agents, especially aspirin (acetylsalicylic acid), are among the most widely prescribed drugs since the long-term use protects against occlusive vascular events [1]. Continued use of antiplatelet medication is high in patients with chronic comorbidities such as cardiovascular diseases, peripheral artery disease, and diabetes [2, 3]. Hence, chronic antiplatelet drug use is frequently observed in septic patients admitted to Intensive Care Units (ICU) [3]. This is of special interest as septic patients with antiplatelet therapy (APT) represent a high-risk population as these patients are older, have more comorbidities, and an augmented disease severity [3, 4]. The impact of antiplatelet agents on coagulation as well as the immune system inter alia by modulation of platelet activation is widely examined [5, 6]. However, due to contradictory studies, the administration of antiplatelet drugs in critically ill patients remains controversial. On the one hand, a continued chronic antiplatelet therapy during the acute phase of sepsis is associated with a survival benefit in sepsis, a lower incidence of acute respiratory distress syndrome (ARDS), and reduced thromboembolic events [7–11]. On the other hand, antiplatelet agents may elevate the risk of bleeding in surgical patients, increase the risk of gastrointestinal bleedings, and may be associated with higher risks of severe sepsis as well as prolonged mechanical ventilation [12–14]. However, data on the need for transfusions in septic patients with continued APT remain scarce. It is recognized that bleeding, transfusion of red blood concentrate (RBC), and platelet transfusions are associated with increased risk for infections and death in the critically ill [15]. We hypothesized that the continuation of a pre-existing APT in septic patients is not associated with higher blood transfusion requirements. Therefore, the need for RBC transfusion served as the primary outcome measure.

## Methods

### Design and study population

The present single centre study was conducted as a secondary analysis of a prospective observational trial that was performed to evaluate the long-term effects of a local continuous sepsis awareness program [16]. The study was performed according to the German data protection rules and the Strengthening the Reporting of Observational Studies in Epidemiology (STROBE) checklist (supplement Table 1). The local ethics committee approved the study and granted a waiver of informed consent (Identifier: BB 133/10). All patients aged ≥ 18 years with severe sepsis and septic shock and a chronic APT enrolled in the primary study were considered for inclusion in the current analysis. All patients were treated at the interdisciplinary ICU of the University Hospital of Greifswald, Germany from January 2010 to December 2013 which represented the period with data on antiplatelet therapy within the original study [16]. The original study investigated the impact of a quality improvement initiative for severe sepsis and septic shock. All data included in the present study consists of patients treated after implementation of the quality improvement. During the original study all ICU patients were screened daily by a constant study team for severe sepsis and septic shock. Hence, patients were screened for a body temperature greater than 38 °C (or less than 36 °C), a heart rate greater than 90 beats per minute, tachypnea (respiratory rate greater than 20 breaths per minute or hyperventilation as indicated by a PaCO_2_ of less than 32 mm Hg), and an alteration in white blood cell count, such as count greater than 12,000/ mm^3^ (or less than 4,000/ mm^3^ or ≥ 10% immature neutrophil granulocytes) in combination with a confirmed or suspected infection as well as organ dysfunction (≥ 1 criteria) which was defined as severe sepsis. Criteria for organ dysfunction were defined as an acutely altered mental state (confusion, agitation, and delirium), thrombocytopenia (decrease of more than 30%/ 24 h or platelets ≤ 100,000 mm^3^), arterial hypoxemia (≤ 75 mm Hg under ambient air or a paO_2_/ FiO_2_ of ≤ 250 mm Hg with oxygen supply), renal dysfunction (diuresis of ≤ 0.5 ml/kg/h for at least 2 h in spite of adequate fluid resuscitation or increase of creatinine greater than double standard value), and metabolic acidosis (base excess ≤ -5 mmol/L or lactate > 3.3 mmol/L. Finally, septic shock was defined as severe sepsis when combined with hypotension (systolic blood pressure of ≤ 90 mm Hg or mean arterial pressure ≤ 65 mm Hg) for at least 1 h despite adequate fluid resuscitation as well as the need for vasopressors to achieve a systolic blood pressure of ≥ 65 mm Hg [16]. Additionally, the present analysis examined and extracted drug administration records and oral administration of APT during intensive care treatment (supplement Table 2). A single drug administration was not considered as continued treatment. All included patients were prospectively followed up to determine baseline, therapeutic and outcome variables. Other patients’ characteristics were determined retrospectively. The indication of transfusing RBC concentrates was made by experienced intensivists in charge, based on objective criteria as well as physiological triggers. The cause of death was determined by scanning the medical records, particularly the death certificate and if available by the results of an autopsy. Patients received sepsis management in accordance with the local institutional sepsis protocol based on the respective Surviving Sepsis Campaign Guidelines [17]. Each individual APT was determined by experienced physicians, considering the patients’ individual risk of thromboembolism and bleeding. Therefore, coronary artery disease and coronary stents were strong indications to continue a pre-existing APT.

**Table 1:** Patient characteristics

	All patients				Matched patients			
Variable	N	Discontinued, *N* = 89	Continued, *N* = 114	*p*-value^c^	N	Discontinued, *N* = 60	Continued, *N* = 60	*p*-value^c^
Male sex, n (%)	203	53 (59.6%)	78 (68.4%)	0.24	120	38 (63.3%)	37 (61.7%)	> 0.99
Age [years], mean (SD)	203	72 (10)	73 (11)	0.77	120	73 (10)	72 (11)	0.60
Septic shock, n (%)	203	71 (79.8%)	86 (75.4%)	0.50	120	46 (76.7%)	46 (76.7%)	> 0.99
SAPS II score, mean (SD)	195	50 (14)	45 (12)	0.017	116	50 (15)	46 (12)	0.18
SOFA, median (IQR)	199	9 (7, 10)	8 (6, 10)	0.087	118	9 (7, 10)	9 (7, 11)	0.93
APACHE II score, mean (SD)	195	22 (7)	21 (7)	0.22	116	22 (7)	21 (7)	0.24
Body temperature [°C], mean (SD)	192	37.01 (1.89)	37.37 (1.72)	0.17	116	37.11 (1.92)	37.11 (1.93)	> 0.99
T at sepsis onset [Gpt/L], median (IQR)	203	191 (133, 272)	230 (170, 327)	0.014	120	201 (147, 296)	214 (144, 303)	0.71
T at ICU discharge [Gpt/L], median (IQR)	200	187 (104, 276)	264 (180, 368)	< 0.001	117	205 (84, 276)	248 (176, 404)	0.034
WBC before sepsis onset [10^9^/L], median (IQR)	183	14 (8, 21)	15 (10, 21)	0.33	111	13 (8, 21)	15 (10, 22)	0.40
Lactate first 24 h after sepsis onset [mmol/l], median (IQR)	192	3.0 (1.7, 6.2)	2.2 (1.5, 4.1)	0.033	117	3.5 (1.6, 6.7)	2.8 (1.7, 4.8)	0.56
CRP first 24 h after sepsis onset [mg/L], median (IQR)	178	210 (118, 270)	201 (117, 285)	0.84	104	205 (127, 270)	186 (114, 274)	0.63
PCT first 24 h after sepsis onset [ng/mL], median (IQR)	166	7 (2, 22)	6 (2, 20)	0.47	99	7 (2, 30)	6 (2, 21)	0.81
Colonization with nosocomial pathogens at admission, n (%)	202	12 (13.6%)	7 (6.1%)	0.089	119	8 (13.6%)	4 (6.7%)	0.24
Hospital-acquired nosocomial pathogen, n (%)	203	15 (16.9%)	16 (14.0%)	0.69	120	11 (18.3%)	8 (13.3%)	0.62
Operation ± 3 days to sepsis onset, n (%)	203	60 (67.4%)	70 (61.4%)	0.46	132	43 (71.7%)	42 (70.0%)	> 0.99
**Reason for pre-existing APT (%)**	203			< 0.001	120			0.58
Primary prevention		23 (25.8%)	8 (7.0%)			9 (15.0%)	6 (10.0%)	
Secondary prevention		66 (74,2%)	106 (93.0%)			51 (85.0%)	54 (90.0%)	
**Comorbidities**								
Arterial hypertension, n (%)	203	73 (82.0%)	86 (75.4%)	0.30	120	48 (80.0%)	48 (80.0%)	> 0.99
Coronary artery disease, n (%)	203	24 (27.0%)	53 (46.5%)	0.006	120	17 (28.3%)	20 (33.3%)	0.69
Atrial fibrillation, n (%)	203	22 (24.7%)	26 (22.8%)	0.87	120	15 (25.0%)	8 (13.3%)	0.16
Myocardial infarction, n (%)	202	15 (16.9%)	34 (30.1%)	0.032	120	12 (20.0%)	13 (21.7%)	> 0.99
Peripheral artery occlusive disease, n (%)	202	18 (20.2%)	37 (32.7%)	0.056	120	17 (28.3%)	19 (31.7%)	0.84
Cerebrovascular disease, n (%)	202	28 (31.5%)	34 (30.1%)	0.88	120	19 (31.7%)	19 (31.7%)	> 0.99
Peptic ulcer disease, n (%)	202	11 (12.4%)	17 (15.0%)	0.68	120	7 (11.7%)	10 (16.7%)	0.60
Other pre-existing chronic disease^a^, n (%)	203	42 (47.2%)	58 (50.9%)	0.67	120	27 (45.0%)	30 (50.0%)	0.71
**Pre-existing long-term treatment**								
Statins, n (%)	203	30 (33.7%)	63 (55.3%)	0.003	120	22 (36.7%)	28 (46.7%)	0.35
Betablockers, n (%)	203	52 (58.4%)	75 (65.8%)	0.31	120	39 (65.0%)	35 (58.3%)	0.57
Antihypertensive drugs^b^, n (%)	203	63 (70.8%)	83 (72.8%)	0.76	120	43 (71.7%)	42 (70.0%)	> 0.99
**Source of sepsis**	196			0.85	116			0.69
Abdominal, n (%)		39 (46.4)	57 (50.9)			27 (47.4)	33 (55.9)	
Pulmonary, n (%)		26 (31.0)	33 (29.5)			18 (31.6)	13 (22.0)	
Bone and soft tissue, n (%)		6 (7.1)	9 (8.0)			4 (7.0)	4 (6.8)	
Other, n (%)		13 (15.5)	13 (11.6)			8 (14.0)	9 (15.3)	

Apache: Acute physiology and chronic health evaluation; APT: antiplatelet therapy; CRP: C-reactive protein; IQR: interquartile range; SAPS: Simplified acute physiology score; SD: Standard deviation; PCT: Procalcitonin; T: Thrombocytes; WBC: White blood cells. ^a^at least one of the listed diseases: chronic kidney failure, metastatic cancer, hematological malignancies, AIDS and other causes of immunosuppression, severe hepatic failure, NYHA class IV and pre-existing chronic severe hypoxia; ^b^at least one of the listed drugs: angiotensin-converting enzyme inhibitor, angiotensin receptor blocker, calcium antagonists, minoxidil, moxonidine, nitrates, and molsidomine; ^c^Fisher’s exact test; Two Sample t-test; Wilcoxon rank sum test

**Table 2:** Coagulation and need of transfusion

	All patients				Matched patients			
Variable	N	Discontinued, *N* = 89	Continued, *N* = 114	*p*-value^1^	N	Discontinued, *N* = 60	Continued, *N* = 60	*p*-value^1^
**Coagulation**								
**Hemoglobin** [mmol/L], median (IQR)								
at sepsis onset	203	6.50 (5.90, 7.90)	6.50 (5.82, 7.65)	0.69	120	6.40 (5.90, 8.03)	6.65 (6.00, 7.80)	0.54
at ICU discharge	200	5.80 (5.30, 6.50)	5.80 (5.40, 6.27)	0.52	117	6.00 (5.40, 6.60)	5.80 (5.40, 6.20)	0.26
**Activated partial thromboplastin** [s], median (IQR)								
at sepsis onset	203	30 (26, 36)	29 (26, 33)	0.30	120	31 (26, 36)	29 (27, 33)	0.48
at ICU discharge	200	32 (27, 38)	30 (26, 37)	0.074	117	31 (27, 37)	30 (26, 40)	0.52
**Prothrombin time** [s], median (IQR)								
at sepsis onset	203	83 (59, 94)	84 (67, 94)	0.17	120	79 (59, 95)	80 (60, 93)	0.91
at ICU discharge	199	87 (67, 98)	94 (82, 103)	0.025	116	84 (64, 97)	95 (81, 104)	0.023
**Need for transfusions**								
**Transfusions required, n (%)**	203	59 (66.3%)	84 (73.7%)	0.28	120	39 (65.0%)	43 (71.7%)	0.56
**Major bleeding events**^2^, n (%)	203	33 (37.1%)	34 (29.8%)	0.30	120	22 (36.7%)	17 (28.3%)	0.44
**Red blood cell concentrates**, n (%)								
during ICU stay after sepsis onset	203	51 (57.3%)	81 (71.1%)	0.054	120	31 (51.7%)	41 (68.3%)	0.093
during ICU stay	203	54 (60.7%)	83 (72.8%)	0.072	120	34 (56.7%)	42 (70.0%)	0.18
during total hospital stay	203	64 (71.9%)	91 (79.8%)	0.24	120	42 (70.0%)	46 (76.7%)	0.54
**Platelet concentrates**, n (%)								
during ICU stay after sepsis onset	203	21 (23.6%)	19 (16.7%)	0.29	120	13 (21.7%)	11 (18.3%)	0.82
during ICU stay	203	25 (28.1%)	20 (17.5%)	0.089	120	15 (25.0%)	12 (20.0%)	0.66
during total hospital stay	203	26 (29.2%)	27 (23.7%)	0.42	120	15 (25.0%)	15 (25.0%)	> 0.99
**Fresh frozen plasma concentrates**, n (%)								
during ICU stay after sepsis onset	203	34 (38.2%)	31 (27.2%)	0.10	120	23 (38.3%)	20 (33.3%)	0.70
during ICU stay	203	36 (40.4%)	34 (29.8%)	0.14	120	25 (41.7%)	21 (35.0%)	0.57
during total hospital stay	203	42 (47.2%)	44 (38.6%)	0.25	120	28 (46.7%)	26 (43.3%)	0.85

IQR: interquartile range; ^1^Fisher’s exact test; Wilcoxon rank sum test; Wilcoxon rank sum exact test, ^2^Bleeding that causes transfusion of > 2 red blood concentrates within +/-3 days to ICU admission

### Study groups and endpoints

The study population was divided into two groups according to the management of the pre-existing antiplatelet therapy during intensive care treatment.

1. Discontinued group: the pre-existing chronic use of antiplatelet agents was discontinued on ICU admission and never re-administered during ICU treatment.
2. Continued group: the pre-existing chronic use of antiplatelet agents was continued or interrupted only briefly during ICU treatment. The primary goal was to investigate the effects of continued vs. discontinued pre-existing APT in ICU patients with severe sepsis and septic shock regarding need for RBC concentrate transfusion. Secondary endpoints included mortality rates after sepsis onset, during hospital and ICU stay as well as lengths of stay. The number of transfused blood products e.g. RBC concentrates, platelet concentrates, fresh frozen plasma concentrates (FFP) were thoroughly collected within both groups, as well as mortality rates up to 90-day survival.

### Statistical analysis

Statistical analyses were conducted with GNU R version 4.3.2 (Language and Environment for Statistical Computing, R Core Team, Foundation for Statistical Computing, Vienna, Austria). Descriptive statistics are provided by counts and percentages for categorical data. Continuous variables were visually assessed for normality and summarized as means with standard deviations or medians with quartiles. Two-tailed P values were computed for comparing discontinued and continued pre-existing APT. Fisher’s exact test was used to compare categorical variables at sepsis onset such as age, sex, reason for pre-existing APT (primary or secondary prevention), pre-existing conditions (arterial hypertension; cardiovascular disease[atrial fibrillation, myocardial infarction, or coronary artery disease], peripheral artery disease), antihypertensive medication, lactate level of the first 24 h after sepsis onset [logarithmized], transfusions before sepsis onset, categorized number of RBC cell concentrates, platelet concentrates, FFP, coagulation at sepsis onset (hemoglobin [logarithmized], activated partial thromboplastine time; aPTT, categorized), and prothrombin time at sepsis onset. Patients in the discontinued and continued group were matched for similar patient characteristics. Matching was performed using age, sex, reason for pre-existing APT, arterial hypertension, cardiovascular disease, peripheral artery disease, antihypertensive medication, platelet count of first 24 h after sepsis onset, hemoglobin at sepsis onset (logarithmic), activated partial thromboplastin time at sepsis onset, prothrombin time at sepsis onset, need for transfusions before sepsis onset, and sepsis severity. The estimate was set to ATT (Average Treatment Effect on the Treated) and the variable for exact matching was sepsis severity according to the Sepsis-2 definition. Therefore, matched patients had to have the same severity of sepsis (i.e. either severe sepsis or septic shock) in addition to similar general characteristics. A caliper value of 0.25 was used as a threshold to control matching pairs. Balancing was achieved in most of the variables using genetic matching with the MatchIt package for R. Covariate balancing is shown in supplement Fig. 1. Survival functions and the number of patients at risk were computed using the Kaplan-Meier estimator. Log-rank tests were computed from all matched samples, matched patients who survived the acute phase (≥ 7 days) and 28-day survivors (supplement Table 3). The latter comparisons exclude short-term effects and an imbalance due to patients dying shortly after ICU admission. Incomplete data was imputed using the Mice package for R utilizing predictive mean matching. The pattern of the missing data concerning the matched samples is depicted in supplement Fig. 2. Since the proportional hazards assumption for the multivariable Cox regression is violated (due to the limited and variable influence of blood parameters at sepsis onset), the time since sepsis onset was categorized at 7 and 28 days and added as interaction factor to the Cox regression (using timeSplitter function for the Greg package for R). Potential risk factors entered together into a time-dependent multivariable Cox proportional hazards regression, which was reduced by a stepwise procedure to its essential set of predictor variable to compensate for overfitting. The goodness-of-fit characteristics of the final time dependent multivariable Cox regression performed on matched samples are provided in supplement Table 4.

**Fig. 1:**
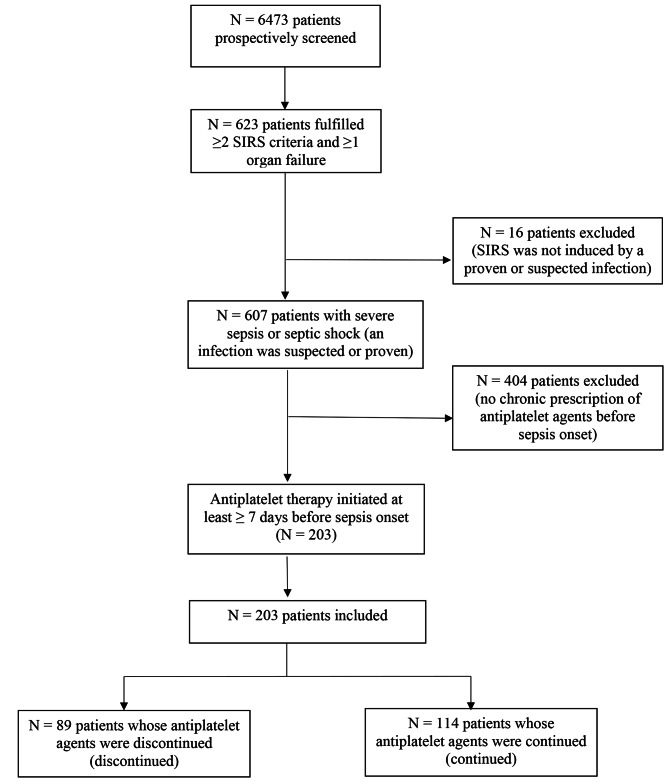
Study population and study flow diagram SIRS: systemic inflammatory response syndrome

**Table 3:** Survival outcome and length of stay

	All patients				Matched patients			
Variable	N	Discontinued, *N* = 89	Continued, *N* = 114	p-value^1^	N	Discontinued, *N* = 60	Continued, *N* = 60	*p*-value^1^
**Survival**								
90-day survival, n (%)	203	34 (38.2%)	76 (66.7%)	< 0.001	120	18 (30.0%)	42 (70.0%)	< 0.001
ICU survival, n (%)	203	52 (58.4%)	91 (79.8%)	0.001	120	35 (58.3%)	48 (80.0%)	0.017
Hospital survival, n (%)	203	43 (48.3%)	85 (74.6%)	< 0.001	120	27 (45.0%)	45 (75.0%)	0.001
**Length of stay**								
*All patients*								
Hospital stay before sepsis onset [days], median (IQR)	203	2 (0, 10)	2 (0, 6)	0.91	120	3 (0, 9)	1 (0, 7)	0.84
ICU stay after sepsis onset [days], median (IQR)	203	6 (2, 14)	12 (6, 24)	< 0.001	120	6 (2, 14)	12 (6, 23)	0.002
Hospital stay [days], median (IQR)	203	21 (7, 49)	29 (17, 55)	0.012	120	21 (4, 46)	29 (15, 52)	0.046
*Survivors of hospital stay*								
Hospital stay before sepsis onset [days], median (IQR)	128	2 (0, 9)	2 (0, 6)	0.85	72	4 (0, 9)	1 (0, 8)	0.64
ICU stay after sepsis onset [days], median (IQR)	128	8 (4, 16)	14 (7, 24)	0.019	72	8 (5, 16)	13 (7, 22)	0.092
Hospital stay [days], median (IQR)	128	28 (18, 50)	32 (20, 56)	0.47	72	28 (16, 46)	30 (20, 52)	0.50

IQR: interquartile range; ^1^Fisher’s exact test; Wilcoxon rank sum test; Wilcoxon rank sum exact test

**Fig. 2:**
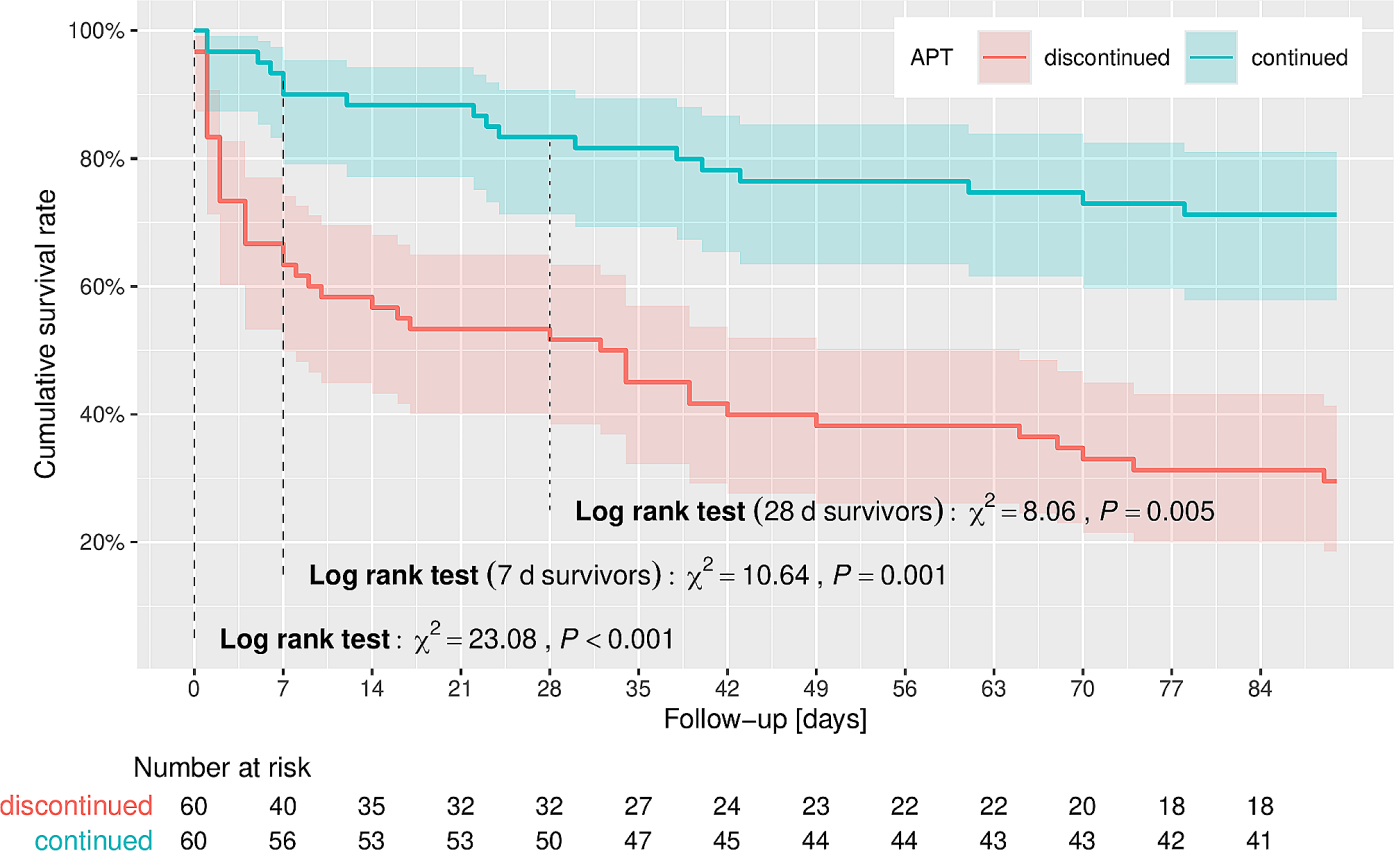
Kaplan-Meier survival estimator of matched patients whose antiplatelet agents were continued or discontinued with 95% confidence bands and log rank test results

## Results

Among the prospectively screened patients (*n* = 6473), 203 had severe sepsis or septic shock as well as a chronic use of antiplatelet agents before sepsis onset and were enrolled in the analysis (Fig. 1). Of these, 31 patients (15.3%) received APT for primary prevention. In 89 patients the pre-existing APT was discontinued whereas it was continued in 114 patients, respectively. The use of aspirin was the most common pre-existing APT (*n* = 180/203 = 88.7%) and most patients with continued APT received aspirin only (supplement Table 2).

### Patients’ characteristics

In both groups of unmatched patients, more male subjects were included (discontinued: 59.6% vs. continued: 68.4%; *p* = 0.24). Mean patient age was similar between both groups (discontinued: 72±10 vs. continued: 73±11 years; *p* = 0.77). Sepsis severity expressed by the sequential organ failure assessment (SOFA) did not differ significantly in unmatched (discontinued: 9 IQR 7 to 10 vs. continued: 8 IQR 6 to 10; *p* = 0.09) as well as matched patients (discontinued: 9 IQR 7 to 10 vs. continued: 9 IQR 7 to 11; *p* = 0.93). Data on mean simplified acute physiology score (SAPS II), APACHE II, and occurrence of septic shock are summarized in Table 1. Moreover, mean body temperature was detected, indicating no significant difference (*p* = 0.17). Besides, data on basic laboratory values were explored, showing higher thrombocyte counts in patients with discontinued treatment, both at sepsis onset (discontinued: 191 IQR: 133 to 272 vs. continued: 230 IQR 170 to 327 gpt/l; *p* = 0.01) as well as at ICU discharge (discontinued: 187 IQR: 104 to 276 vs. continued: 264 IQR 180 to 368 gpt/l; *p* < 0.01) and no significant effect on white blood cell count (*p* = 0.33). Similarly, arterial lactate values were significantly lower in patients with continued APT treatment (discontinued: 3.0 IQR: 1.7 to 6.2 vs. continued: 2.2 IQR 1.5 to 4.1 mmol/l; *p* = 0.03). Additionally, C-reactive protein, procalcitonin, colonization with nosocomial pathogens at admission, hospital-acquired nosocomial pathogens, source of sepsis, and operations within 3 days to sepsis onset were evaluated, all indicating no statistically significant difference between both study groups of unmatched patients (all *p* > 0.05). Furthermore, data on comorbidities were collected (Table 1). Apart from coronary artery disease (*p* < 0.01) and myocardial infarction (*p* = 0.03), no significant differences were noted concerning arterial hypertension, atrial fibrillation, peripheral artery disease, cerebrovascular disease, peptic ulcer disease, and other pre-existing chronic diseases (all *p* > 0.05). Considering pre-existing long-term treatment, statins were used significantly more frequently (*p* < 0.01) in the continued group whereas betablockers and antihypertensive drugs were not (*p* > 0.05). Moreover, data on the indication of the APT were collected. Interestingly, significantly more unmatched patients received APT for primary prevention in the discontinued group (*p* < 0.01). Accordingly, 60 patients in each study arm were matched and balanced for comorbidities, pre-existing long-term treatment, SOFA score, indication for APT, and most conditions at sepsis onset as presented in Table 1. After matching, thrombocyte count was significantly higher in the continued treatment group at ICU discharge (*p* = 0.03). Other factors, particularly those describing severity of sepsis, are balanced in the matched cohort (Table 1 and supplement Fig. 1). Comprehensive characteristics of all and matched patients are provided in Table 1.

### Need for transfusion

We did not detect a significant difference regarding the need for transfusion of RBC concentrates during ICU stay after sepsis onset (Table 2) in unmatched (discontinued: 57.3% vs. continued: 71.1%; *p* = 0.054) and matched (discontinued: 51.7% vs. continued: 68.3%; *p* = 0.09) patients as well as during the total hospital stay (both *p* > 0.05). Similarly, we did not detect a significant difference regarding platelet concentrates in unmatched and matched patients (*p* > 0.05). Finally, the need for FFP concentrate transfusion did not differ significantly, neither in unmatched nor matched patients at all available time periods (all *p* > 0.05).

### Intensive care treatment

Standard intensive care treatment did not differ significantly between both groups such as blood culture sampling, crystalloids, pre-existing antibiotic therapy (before sepsis onset), and catecholamine administration (all *p* > 0.05). Therapeutic variables are depicted in supplement Table 5. When coagulation characteristics were assessed, activated thromboplastin as well as prothrombin time at sepsis onset did not differ significantly whereas the prothrombin time on ICU discharge was significantly lower in unmatched patients with discontinued treatment (*p* = 0.02). Consistently, the number of major bleeding events was not increased in unmatched as well as matched patients with continued APT (*p* > 0.05; Table 2).

### Mortality, length of stay and survival analysis

Length of stay (LOS) on ICU after sepsis onset (*p* < 0.01) and hospital LOS (*p* = 0.01) were prolonged in the continued APT group (unmatched patients). Also, 90-day survival (38.2% vs. 66.7%; *p* < 0.001) as well as ICU (58.4% vs. 79.8%; *p* = 0.001) and hospital survival (48.3% vs. 74.6%; *p* < 0.001) were significantly higher within the continued group (unmatched patients, Table 3). These results are supported after the propensity score matching as survival (90-days, ICU, hospital) remained significantly higher in patients with continued treatment (all *p* < 0.05). Consistently, LOS on ICU after sepsis onset and hospital LOS were prolonged in the continued group (both *p* < 0.01). The Log-rank test confirmed an association between a continuation of chronic APT and survival in the overall study population (*p* < 0.001). Even after exclusion of patients that died within the acute phase of sepsis (first 7 days after sepsis onset) this association was present in matched patients (*p* = 0.001; Fig. 2).

## Discussion

In this secondary analysis we did not observe a significantly increased need for RBC concentrate transfusions in septic patients where APT was continued. This effect was examined in matched (*n* = 120) as well as unmatched patients (*n* = 203). Of note, despite being not significant, a higher number of RBC concentrate transfusions were examined in matched as well as unmatched patients with continued APT. In this context, the level of hemoglobin at sepsis onset as well as on ICU discharge was comparable between both groups. This is important as transfusion triggers might have been similar despite pronounced coagulation derangements within the discontinued group at sepsis onset. A prolonged activated PTT (partial thromboplastin time) is known to be an independent predictor of major bleeding in ICU patients [18]. On first sight, using effective anticoagulants/ APT in septic patients who often present with coagulation derangements and clinical obvious coagulopathy appears unfavorable [18, 19]. However, in the present study there was no significantly increased need for RBC transfusion in continued APT which represents an important finding supporting evidence to continue APT in selected patients. Interestingly, plasmatic coagulation indicators (activated partial thromboplastin time and prothrombin time) were significantly diminished within the discontinued study group. These derangements might be caused by the enhanced sepsis severity. In this sense, the number of FFP concentrates was not significantly impacted by continuation of APT despite a higher number of FFP transfusions in patients with discontinued APT. In this context, the possibility of potential selection bias must be considered. Of note, significantly more patients within the discontinued group had APT for primary prevention. This may have led the intensivist in charge to discontinue APT in those patients more frequently. As a result, sensitivity analysis included the indication for APT. Also, it cannot be excluded that patients with a higher risk of bleeding had both platelet concentrates as well as the agent ceased. In this case, these patients might indicate a different risk profile rather than a comparator group. In addition, the number of severe bleeding events was not affected and patients in need of surgical procedures (within 3 days of sepsis onset), e.g. for source control, who had a particularly high risk for bleeding complications, were equally represented in both study groups. As abdominal infection is the leading cause of sepsis in our study, most of all patients had surgery during the acute phase of sepsis (67.4% vs. 61.4%; *p* = 0.46). In conclusion, our study supports the evidence for continuation of APT in this population.

The findings appear to be at odds with existing research regarding the ongoing administration of antiplatelet agents prior to elective surgery. The administration of aspirin before surgery and throughout the early postsurgical period in patients with an elevated risk for vascular complications had no effect on thromboembolic events but increased the risk of bleeding [12]. A meta-analysis has confirmed that aspirin is linked to a heightened incidence of major perioperative haemorrhages, thereby questioning the appropriateness of maintaining aspirin therapy in patients with intermediate-risk cardiovascular conditions [20]. From our point of view these results might not be applicable to the present study population since preoperative sepsis is an independent risk factor for postoperative arterial and venous thromboses increasing with the severity of sepsis [21].

When examining the potential impact of APT on mortality rates, the continued use of chronic APT was found to correlate with improved survival rates at 90 days, as well as enhanced survival rates in both ICU and hospital settings. This potential linkage aligns with recent research indicating reduced mortality rates among septic patients who were treated with uninterrupted APT [7–9]. This is of particular interest, as recent investigations, such as the ANTISEPSIS study, have reported no diminution in sepsis among elderly patients within a cohort utilizing aspirin for primary prevention. This reinforces the prevailing uncertainty within the scientific community [22]. Mortality represents an objective endpoint and is easily measurable. However, it is very difficult to attribute the effects on mortality to a single intervention as even in the matched group small differences such in the SAPS II scores may indicate different risk profiles, which may have affected the mortality rates considerably. In order to clarify the severity of sepsis among study groups, the SOFA score was evaluated. Of note, both matched and unmatched patients did not differ significantly in their SOFA score. Furthermore, despite adjustments for age, the indication for APT, and lactate level, it could be contended that patients in the discontinued group were inherently at a higher risk of mortality, thereby complicating the interpretation of the findings. As indicated by the SAPS II score, patients in the discontinued group had a higher disease severity. This is reflected by the magnitude of the detected effect. A more than 25% absolute risk reduction for 90-day mortality seems to be high and might be related to either underlying differences within the patient population or due to effects of other covariates associated with APT continuation [23].

The inflammation mediated activation of platelets that interact with leucocytes, endothelium, subsequent crosslink inflammation, and coagulation is one of the most important steps in the pathogenesis of sepsis [5, 24–26]. By recruiting neutrophils and an increased occurrence of circulating platelet-leucocyte aggregates, platelet activation contributes to the formation of microthrombi, organ hypoperfusion and thus resulting in multi-organ failure and poor outcomes in septic patients [24, 27]. In this context, a pilot study investigated the potential effects of low-dose acetylsalicylic acid in ICU patients with systemic inflammation without pre-existing APT in a randomized, placebo-controlled setting [28]. The study showed no significant changes in inflammatory biomarkers in patients treated with low-dose acetylsalicylic acid. In contrast, increased concentrations of specific anti-inflammatory lipid mediators were observed [28]. Further investigations outside the more commonly measured cytokine system may further elucidate this topic. Also, sepsis is associated with a transient increased risk of cardiovascular events e.g., myocardial infarction and antiplatelet agents may prevent these thromboembolic complications [29, 30]. In the present study, patients with a continued APT tended to have an elevated risk for thromboembolic complications since a prior history of coronary artery disease or myocardial infarction was almost twice as high as in the discontinued group and significantly more patients received APT for primary prevention in the discontinued group. Consistently, a pre-existing therapy with statins was more frequent within the continued study population. This may have led the intensivist in charge to a continuation of APT representing a mandatory decision in everyday practice. As the risk of thromboembolic events increases with the sepsis severity, patients with a discontinued APT and increased disease severity (higher SAPS II scores, a reduced number of thrombocytes on sepsis onset, and higher lactate levels) had an elevated risk of septic mediated thromboembolic complications. One might speculate that hypercoagulation after cessation of antiplatelet drugs may amplify the septic mediated prothrombotic state leading to an adapted anticoagulative therapy in septic patients.

In this study aspirin was by far the most used antiplatelet drug before and during sepsis. Therefore, no conclusion concerning a drug dependent effect or whether a combination of antiplatelet drugs show similar effects can be made. However, similar results are reported for clopidogrel that also suppresses systemic inflammation [7, 31]. Whether a combined therapy of antiplatelet agents shows beneficial effects upon mortality despite increased bleeding risks and harmful effects due to weakening of platelet-neutrophil-endothelial interactions remains controversial as well [7, 32–34].

Due to an increased number of survived days, the ICU as well as the hospital LOS were significantly increased in patients where APT had been continued. In line with comparable retrospective studies, the present analysis suggests an effect for the total study population if a chronic APT was continued in ICU patients with severe sepsis or septic shock [4, 7, 9]. Furthermore, time-dependent Cox regression analysis confirmed that a continued therapy with antiplatelet agents was associated with an improved short- and long-term survival. We hypothesize that the long-term effects of an APT, most notably the prevention of thromboembolic events, may impact these results instead of supposed short-term immuno-modulating effects. However, the individual effects of antiplatelet agents on coagulation and the immune system are subject to a large inter-individual variability [35].

This study has limitations that must be considered. First, the non-randomized observational and retrospective nature of this single centre study precludes to establish or generalize the causality of the correlation especially as the cause of disruption of a pre-existing APT could not be evaluated retrospectively. Secondly, the pre-existing use of antiplatelet agents was derived from medical records and data on rigorous assessment of adherence to medication were not available. A single dose of APT was counted as discontinued treatment which represents another potential limitation. Self-pay prescription or adherence to prescribed medication cannot be guaranteed. Thus, there might be some misclassifications. Thirdly, due to the clinical approach of the present study, antiplatelet agents in ICU patients were considered individually without strict therapeutic standards. This may have led to variation of mortality rates due to different risk profiles and baseline imbalances that might have not been ruled out by the sensitivity analysis. Hence, the continued APT group consisted of patients with severe cardiovascular disease as well as higher rates of statins in this population. Also, mortality as a primary outcome can be biased towards family decisions as this outcome can be severely impacted by end-of-life decisions which might impact LOS as well [22]. Moreover, even following adjustments for factors such as age, indications for APT, and lactate levels, it could be posited that subjects in the discontinued cohort inherently possessed a higher risk of mortality, thereby muddying the interpretative clarity of the outcomes. Although, individual studies suggest decreased mortality rates in septic patients related to statin therapy [36, 37], evidence from meta-analysis indicated no support for this effect [38]. The indication for transfusing RBC concentrates was made by experienced intensivists in charge, based on objective criteria as well as physiological triggers which represents clinical practise. Against the background of missing evidence, no predefined protocol was followed, and the individual indication of transfusion may have differed based on the physician in charge. In order to overcome potential bias we used a matching procedure to estimate the effects of continued APT on survival. However, we cannot exclusively attribute these results to antiplatelet agents as we cannot rule out other impacting factors due to the retrospective nature of the study. Finally, this study is a secondary analysis and not primarily designed to evaluate the effects of continuation or discontinuation of APT. Future prospective studies including larger numbers of patients are needed to evaluate these effects.

## Conclusion

In conclusion, our secondary analysis revealed no significant increase in the requirement for RBC transfusion among septic patients who continued receiving chronic APT, predominantly aspirin. Furthermore, it hinted a potential link between ongoing APT and enhanced survival rates in these septic patients. A time-dependent Cox regression model adjusted for the primary variables linked with survival was used on matched patients to mitigate potential indication/inclusion biases thereby reinforcing the robustness of the multivariable analysis. Further research should delve deeper into whether the continuation of APT leads to an increased requirement for RBC transfusions, utilizing a randomized controlled approach with a substantially larger patient cohort.

### Electronic supplementary material

Below is the link to the electronic supplementary material.

Supplementary Material 1

## Data Availability

Data and material are available on reasonable request. Inquiries can be sent to sean.scholz@evkb.de.
