# Supplementary material for: Continuation of chronic antiplatelet therapy is not associated with increased need for transfusions: a cohort study in critically ill septic patients
Source: BMC Anesthesiol. 2024 Apr 17;24:146. doi: 10.1186/s12871-024-02516-7 (PMC11022363; doi:10.1186/s12871-024-02516-7)
Supplement: Supplementary file 1 — Supplementary Material 1 [file 12871_2024_2516_MOESM1_ESM.docx]

**Supplement:**

**Supplement table 1. STROBE checklist of items that should be included in reports of observational studies**

|  | Item No. | Recommendation | |  | Relevant text from manuscript |
| --- | --- | --- | --- | --- | --- |
| **Title and abstract** | 1 | (*a*) Indicate the study’s design with a commonly used term in the title or the abstract |  | | (…) a cohort study in critically ill septic patients.  (…) a secondary analysis of a prospective cohort study. |
|  |  | (*b*) Provide in the abstract an informative and balanced summary of what was done and what was found |  | | This information is provided where appropriate. |
| Introduction | | | | |  |
| Background/rationale | 2 | Explain the scientific background and rationale for the investigation being reported |  | | This information is provided in the Introduction. |
| Objectives | 3 | State specific objectives, including any prespecified hypotheses |  | | We hypothesized that the continuation of a pre-existing APT in septic patients may not affect the need for transfusion which served as the primary outcome measure. |
| Methods | | | | |  |
| Study design | 4 | Present key elements of study design early in the paper |  | | This information is provided early in the Design and study population section. |
| Setting | 5 | Describe the setting, locations, and relevant dates, including periods of recruitment, exposure, follow-up, and data collection |  | | Patients treated at the interdisciplinary ICU of the University Hospital of Greifswald (setting/ location) (…) from January 2010 to December 2013. (relevant dates/ recruitment), Not applicable (exposure), All included patients were prospectively followed up to determine baseline, therapeutic and outcome variables (follow-up, and data collection). |
| Participants | 6 | (*a*) *Cohort study*—Give the eligibility criteria, and the sources and methods of selection of participants. Describe methods of follow-up |  | | All patients aged ≥ 18 years with severe sepsis and septic shock and a chronic APT enrolled in the primary study were considered for inclusion in the current analysis. All patients were treated at the interdisciplinary ICU of the University Hospital of Greifswald, Germany from January 2010 to December 2013 which represented the period with data on antiplatelet therapy within the original study. The original study investigated the impact of a quality improvement initiative for severe sepsis and septic shock. All data included in the present study consists of patients treated after implementation of the quality improvement. During the original study all ICU patients were screened daily by a constant study team for severe sepsis and septic shock. |
| Variables | 7 | Clearly define all outcomes, exposures, predictors, potential confounders, and effect modifiers. Give diagnostic criteria, if applicable |  | | The primary goal was to investigate the effects of continued vs discontinued pre-existing APT in ICU patients with severe sepsis and septic shock regarding need for RBC concentrate transfusion. Secondary endpoints included mortality rates after sepsis onset, during hospital and ICU stay as well as lengths of stay. The number of transfused blood products (RBC concentrates, platelet concentrates, fresh frozen plasma concentrates) were thoroughly collected within both groups, as well as mortality rates up to 90-day survival. |
| Data sources/ measurement | 8 | For each variable of interest, give sources of data and details of methods of assessment (measurement). Describe comparability of assessment methods if there is more than one group |  | | All data included in the present study consist of patients treated after implementation of the quality improvement. During the original study all ICU patients were screened daily by a constant study team for severe sepsis and septic shock. Exact definitions were previously described in Scheer et al. Additionally, the present analysis examined drug administration records and oral administration of APT during intensive care treatment was extracted (measurement): Not applicable; (assessment methods): Not applicable. |
| Bias | 9 | Describe any efforts to address potential sources of bias |  | | Different statistical approaches were used in order to address potential bias (e.g. propensity score matching, time-dependent Cox regression model adjusted for the major variables that are associated with survival). The goodness-of-fit characteristics of the final time dependent multivariable Cox regression performed on matched samples is provided in supplement table 4. |
| Study size | 10 | Explain how the study size was arrived at |  | | Among the prospectively screened patients (n=6473), 203 had severe sepsis or septic shock as well as a chronic use of antiplatelet agents before sepsis onset and were enrolled in the analysis (Figure 1). In 89 patients the pre-existing APT was discontinued whereas it was continued in 114 patients, respectively. |

| Quantitative variables | 11 | Explain how quantitative variables were handled in the analyses. If applicable, describe which groupings were chosen and why | Descriptive statistics are provided by counts and percentages for categorical data. Numerical data are expressed by means and standard deviations or median with quartile ranges as appropriate. |  |
| --- | --- | --- | --- | --- |
| Statistical methods | 12 | (*a*) Describe all statistical methods, including those used to control for confounding | Statistical analyses were conducted with GNU R version 4.3.2 (Language and Environment for Statistical Computing, R Core Team, Foundation for Statistical Computing, Vienna, Austria). Descriptive statistics are provided by counts and percentages for categorical data. Numerical data are expressed by means and standard deviations or median with quartile ranges as appropriate. Two-tailed P values were computed for comparing discontinued and continued pre-existing APT. Precisely Fisher’s exact test was used to compare categorical variables at sepsis onset such as age, sex, reason for pre-existing APT, pre-existing conditions (arterial hypertension; cardiovascular disease [atrial fibrillation, myocardial infarction, or coronary artery disease], peripheral artery disease), antihypertensive medication, lactate level of the first 24 hours after sepsis onset [logarithmized], transfusions before sepsis onset, categorized number of RBC cell concentrates, platelet concentrates, fresh frozen plasma concentrates (FFP), coagulation at sepsis onset (hemoglobin [logarithmized], activated partial thromboplastine time; aPTT, categorized), and prothrombin time at sepsis onset. Sepsis severity [Sepsis-2 definition: severe sepsis / septic shock] was set as the variable for exact matching. Balancing was achieved in most of the variables using genetic matching with the MatchIt package for R. Covariate balancing is shown in supplemental figure 1. Survival functions and the number of patients at risk were computed using the Kaplan-Meier estimator. Log-rank tests were computed from all matched samples, matched patients who survived the acute phase (≥7 days) and 28-day survivors. The latter comparisons do exclude short-term effects and an imbalance due to patients dying shortly after ICU admission. |  |
|  |  | (*b*) Describe any methods used to examine subgroups and interactions | Please see above. |  |
|  |  | (*c*) Explain how missing data were addressed | Incomplete data was imputed using the Mice package for R utilizing predictive mean matching. Since the proportional hazards assumption for the multivariable Cox regression is violated (due to the limited and variable influence of blood parameters at sepsis onset), the time since sepsis onset was categorized at 7 and 28 days and added as interaction factor to the Cox regression (using timeSplitter function for the Greg package for R). The pattern of the missing data concerning the matched samples is depicted in supplement figure 2. |  |
|  |  | (*d*) *Cohort study*—If applicable, explain how loss to follow-up was addressed  *Case-control study*—If applicable, explain how matching of cases and controls was addressed  *Cross-sectional study*—If applicable, describe analytical methods taking account of sampling strategy | Not applicable. |  |
|  |  | (*e*) Describe any sensitivity analyses | Please see section 9. |  |
| Results | | | | |
| Participants | 13* | (a) Report numbers of individuals at each stage of study—eg numbers potentially eligible, examined for eligibility, confirmed eligible, included in the study, completing follow-up, and analysed | Please see figure 1. 6474 patients were screened as described previously. Of these 607 patients had sepsis or septic shock (16 were excluded). All of the records were screened for antiplatelet therapy initiated at least 7 days before sepsis onset leading to 203 patients. (404 patients were excluded because they did not have any antiplatelet agents before sepsis onset). |  |
|  |  | (b) Give reasons for non-participation at each stage | Not applicable. |  |
|  |  | (c) Consider use of a flow diagram | (figure 1) |  |
| Descriptive data | 14* | (a) Give characteristics of study participants (eg demographic, clinical, social) and information on exposures and potential confounders | Comphrehensive data on patient characteristics are provided in table 1. |  |
|  |  | (b) Indicate number of participants with missing data for each variable of interest | The pattern of the missing data concerning the matched samples is depicted in supplement figure 2. The number of missing values can be read from the column N as indicated in the tables such as table 1, 2 or supplement table 2. |  |
|  |  | (c) *Cohort study*—Summarise follow-up time (eg, average and total amount) | Follow-up was performed until day 90. |  |
| Outcome data | 15* | *Cohort study*—Report numbers of outcome events or summary measures over time | Table 2, 3. |  |
| Main results | 16 | (*a*) Give unadjusted estimates and, if applicable, confounder-adjusted estimates and their precision (eg, 95% confidence interval). Make clear which confounders were adjusted for and why they were included | All applicable results are presented in this fashion. |  |
|  |  | (*b*) Report category boundaries when continuous variables were categorized | These are reported where appropriate. |  |
|  |  | (*c*) If relevant, consider translating estimates of relative risk into absolute risk for a meaningful time period | This was considered where appropriate. |  |

| Other analyses | 17 | Report other analyses done—eg analyses of subgroups and interactions, and sensitivity analyses | As stated previously. |
| --- | --- | --- | --- |
| Discussion | | | |
| Key results | 18 | Summarise key results with reference to study objectives | These results suggest that continued antiplatelet treatment did not increase the need for transfusions and suggest a potential association with survival in septic patients. |
| Limitations | 19 | Discuss limitations of the study, taking into account sources of potential bias or imprecision. Discuss both direction and magnitude of any potential bias | Potential limitations are discussed in detail in the limitations section. |
| Interpretation | 20 | Give a cautious overall interpretation of results considering objectives, limitations, multiplicity of analyses, results from similar studies, and other relevant evidence | This was cautiously done in the discussion as well as the limitation section. |
| Generalisability | 21 | Discuss the generalisability (external validity) of the study results | Future studies should further explore whether continuing APT increases RBC transfusions in a randomized controlled fashion with a significantly larger number of patients with regards to the limitations due to our study type. |
| Other information | |  | |
| Funding | 22 | Give the source of funding and the role of the funders for the present study and, if applicable, for the original study on which the present article is based | No funding applicable. |

**Supplement table 2. Antiplatelet drug use before and during intensive care treatment**

| **Discontinued group** |  |
| --- | --- |
|  | chronic administration |
|  |  |
| ASA | 73 |
| ASA + Clopidogrel | 2 |
| ASA + Dipyridamol | 4 |
| Clopidogrel | 10 |

| **Continued group** |  | | | | |
| --- | --- | --- | --- | --- | --- |
|  | chronic administration | continued  administration | | | |
|  |  | ASA | ASA + Clopidogrel | Clopidogrel | Iloprost |
| ASA | 96 | 87 | 8 | 1 | 0 |
| ASA + Clopidogrel | 5 | 2 | 3 | 0 | 0 |
| Clopidogrel | 11 | 4 | 1 | 5 | 1 |
| Dipyridamol | 1 | 1 | 0 | 0 | 0 |
| Dabigatran | 1 | 1 | 0 | 0 | 0 |

**Supplement table 3. Effects of predictors in time-dependent multivariable Cox regression analysis performed on matched samples**

| **Predictor** | **HR (95%-CI)**^1^ | **p-value** |
| --- | --- | --- |
| **Age at sepsis onset** [10-year increase] | | |
| <7 days | 1.70 (1.06 to 2.73) | 0.028 |
| ≥7 days | 2.29 (1.08 to 4.83) | 0.031 |
| ≥28 days | 2.24 (1.28 to 3.92) | 0.006 |
| **Lactate first 24h after sepsis onset** [increase on log2-scale] | | |
| <7 days | 2.56 (1.76 to 3.73) | <0.001 |
| ≥7 days | 1.71 (0.87 to 3.35) | 0.12 |
| ≥28 days | 0.93 (0.59 to 1.47) | 0.74 |
| **Hemoglobin at sepsis onset** [increase on log2-scale] | | |
| <7 days | 2.27 (0.67 to 7.73) | 0.18 |
| ≥7 days | 1.71 (0.87 to 3.35) | 0.20 |
| ≥28 days | 0.93 (0.59 to 1.47) | 0.74 |
| **Colonization with nosocomial pathogens at admission or hospital-acquired nosocomial pathogen** [reference: not present] | | |
| <7 days | 0.81 (0.31 to 2.10) | 0.65 |
| ≥7 days | 3.55 (0.96 to 13.1) | 0.057 |
| ≥28 days | 3.25 (1.21 to 8.70) | 0.020 |
| **Continuation of antiplatelet agents** [reference: discontinued] | | |
| <7 days | 0.24 (0.10 to 0.63) | 0.004 |
| ≥7 days | 0.41 (0.11 to 1.58) | 0.19 |
| ≥28 days | 0.20 (0.07 to 0.57) | 0.003 |

^1^ HR = Hazard ratio, CI = Confidence interval

**Supplement table 4. Goodness-of-fit characteristics of the final time-dependent multivariable Cox regression performed on matched samples**

| **Goodness-of-fit characteristic/test** | **Value**^1^ | **p-value** |
| --- | --- | --- |
| Harrell’s c (concordance) | c=0.833 (SE=0.029) | <0.001 |
| Overall likelihood ratio | 90.52 | <0.001 |
| Score test | 96.05 | <0.001 |
| Wald test | 78.77 | <0.001 |

^1^Average of 10 imputed data sets.

**Supplement table 5. Therapeutic variables**

|  |  | **Antiplatelet therapy** | |  |
| --- | --- | --- | --- | --- |
| **Variable** | **N** | **Discontinued**  N = 89 | **Continued** N = 114 | **p-value^1^** |
| **Operation ±3 days to sepsis onset**, n (%) | 203 | 60 (67.4%) | 70 (61.4%) | 0.46 |
| **Blood culture sampling before antibiotic therapy, n (%)** | 203 |  |  | 0.46 |
| no |  | 64 (71.9%) | 73 (64.0%) |  |
| yes, no pathogen detected |  | 16 (18.0%) | 28 (24.6%) |  |
| yes, pathogen detected |  | 9 (10.1%) | 13 (11.4%) |  |
| **Blood culture sampling after initiation of antibiotic therapy, n (%)** | 203 |  |  | 0.46 |
| no |  | 27 (30.3%) | 28 (24.6%) |  |
| yes, no pathogen detected |  | 42 (47.2%) | 64 (56.1%) |  |
| yes, pathogen detected |  | 20 (22.5%) | 22 (19.3%) |  |
| **Microbiological samples of the septic focus, n (%)** | 202 |  |  | 0.52 |
| no |  | 25 (28.4%) | 26 (22.8%) |  |
| yes, no pathogen detected |  | 16 (18.2%) | 18 (15.8%) |  |
| yes, pathogen detected |  | 47 (53.4%) | 70 (61.4%) |  |
| **Crystalloids first 6h after sepsis onset, n (%)** | 203 |  |  | 0.52 |
| <1 liter |  | 20 (22.5%) | 33 (28.9%) |  |
| 1-2 liter |  | 27 (30.3%) | 35 (30.7%) |  |
| >2 liter |  | 42 (47.2%) | 46 (40.4%) |  |
| **Crystalloids first 24h after sepsis onset, n (%)** | 203 |  |  | 0.57 |
| <3 liter |  | 23 (25.8%) | 36 (31.6%) |  |
| 3-6 liter |  | 32 (36.0%) | 34 (29.8%) |  |
| >6 liter |  | 34 (38.2%) | 44 (38.6%) |  |
| **Preexisting antibiotic therapy (before sepsis onset), n (%)** | 201 | 38 (42.7%) | 59 (52.7%) | 0.20 |
| **Use of dobutamine, n (%)** | 203 | 17 (19.1%) | 27 (23.7%) | 0.49 |
| **Use of low-dose steroids, n (%)** | 203 | 27 (30.3%) | 31 (27.2%) | 0.64 |
| **Use of epinephrine, n (%)** | 203 | 4 (4.5%) | 5 (4.4%) | >0.99 |
| **Use of norepinephrine, n (%)** | 203 | 84 (94.4%) | 102 (89.5%) | 0.31 |
| **Antimycotic therapy, n (%)** | 203 | 29 (32.6%) | 36 (31.6%) | 0.88 |

^1^Fisher's exact test.

**Supplement figure 1. Covariate balance of matched samples**


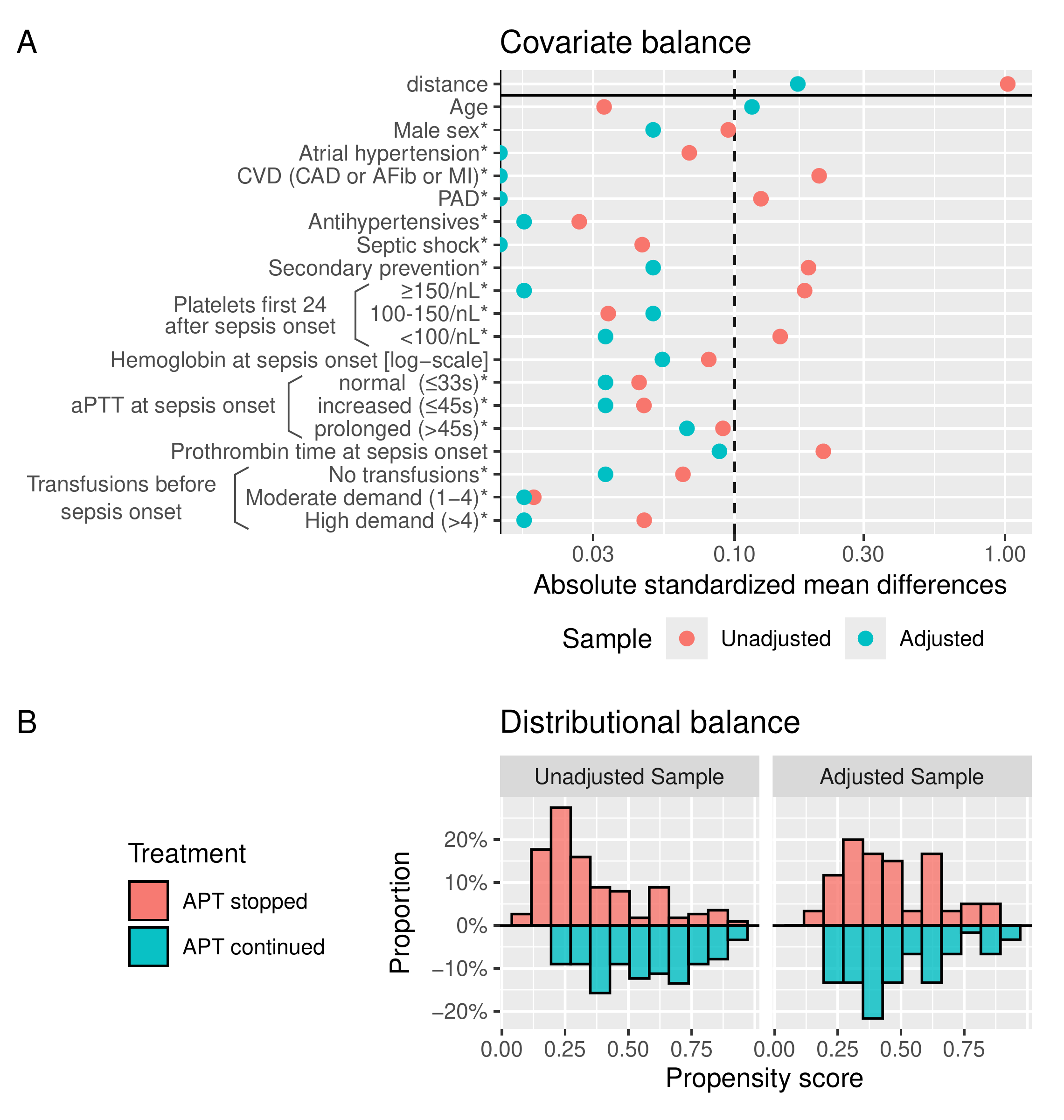


Covariate balance of matched samples (A) and distributional balance for propensity score (B). Matching variables were age, sex, arterial hypertension, Cardiovascular disease (CVD), peripheral artery disease (PAD), antihypertensive medication, platelet counts at sepsis onset categorized for partial SOFA scores, hemoglobin at sepsis onset (logarithmic), activated partial thromboplastin (aPTT) group at sepsis onset, prothrombin time at sepsis onset, need for transfusions before sepsis onset, sepsis severity, and reason for pre-existing APT. The estimand was set to ATT (Average Treatment Effect on the Treated) and sepsis severity was set as variable for exact matching. A caliper value of 0.25 was used as a threshold to control matching pairs; CAD: Coronary artery disease; AFib: Atrial fibrillation; MI: Myocardial infarction.

**Supplement figure 2. Missing data pattern of matched samples**


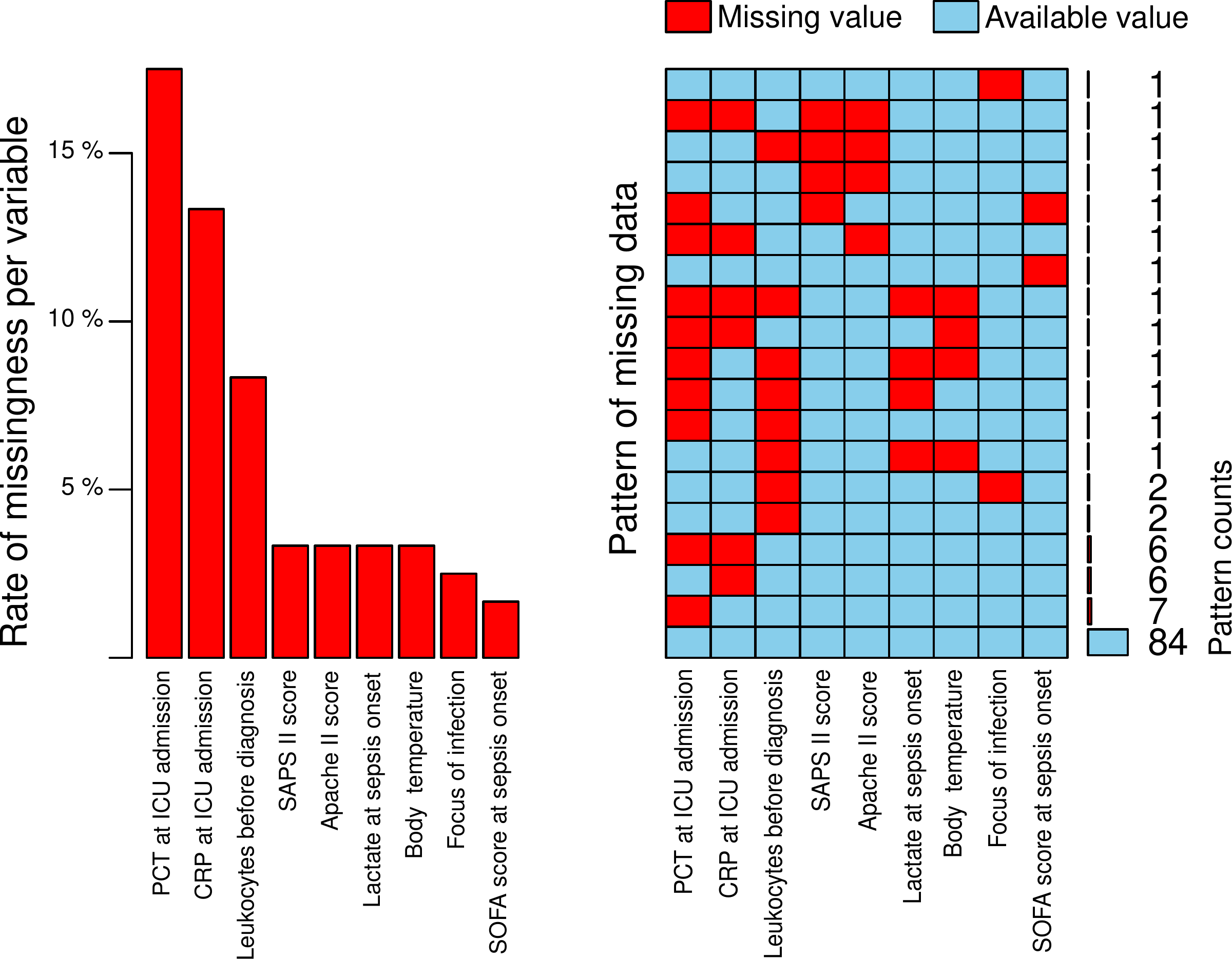


Bar plot of missing data and missing pattern of matched samples that needed to be imputed through predictive mean matching.
